# A patient with a Berger’s space filled by silicone oil

**DOI:** 10.1186/s12886-023-03035-8

**Published:** 2023-07-10

**Authors:** Shuai He, Zenan Lin, Qinghua Qiu

**Affiliations:** 1 Department of Ophthalmology, Shanghai General Hospital, Shanghai Jiao Tong University School of Medicine, Shanghai, PR China; 2 National Clinical Research Center for Eye Diseases, Shanghai Key Laboratory of Ocular Fundus Diseases, Shanghai Engineering Center for Visual Science and Photomedicine, Shanghai Engineering Center for Precise Diagnosis and Treatment of Eye Diseases, Shanghai, PR China; 3 Department of Ophthalmology, Tong Ren Hospital, Shanghai Jiao Tong University School of Medicine, No. 1111 Xianxia Road, Changning District, Shanghai, PR China

**Keywords:** Berger's space, Silicone oil, PPV, Retinal detachment

## Abstract

**Background:**

To report a case in which silicone oil accidentally entered Berger’s space (BS) after vitrectomy and to explore the effective treatments and possible etiological mechanisms.

**Case presentation:**

A 68-year-old male underwent vitrectomy and silicone oil injection for the treatment of retinal detachment (RD) in the right eye. Six months later, we noticed an unexpected lens-like round translucent substance located behind the posterior lens capsule and diagnosed it as BS filled by silicone oil. Subsequently, we conducted vitrectomy and the drainage of the silicone oil in BS in the second surgery. A 3-month follow-up showed significant anatomic recovery and visual recovery.

**Conclusions:**

Our case report presents a patient with silicone oil entering BS after vitrectomy and provides photographs of BS from a relatively unique perspective. Furthermore, we illustrate the surgical treatment procedure and reveal the possible etiology and prevention method of silicon oil entering BS, which will provide good insights for clinical diagnosis and treatment.

**Supplementary Information:**

The online version contains supplementary material available at 10.1186/s12886-023-03035-8.

## Background

In 1887, the Austrian ophthalmologist Émile Berger first described Berger’s space (BS) as a space located between the lens capsule and anterior hyaloids [1]. It was said to be surrounded by the vitreous-lens attachment called Wieger’s ligament. On the coronal plane, the outer circumference limit of the vitreous-lens contact area was named as Egger’s line. Although first reported in the eighteenth century, its existence was confirmed accidently in a few studies. Specifically, the accumulation of hemorrhage, fluid and pigment in BS caused by trauma, infusion misdirection syndrome and pigment dispersion syndrome has recently been reported in the literatures [2–4].

Due to the tight connection of Weiger's ligament and its surrounding tissues under normal conditions, the presence of BS is often overlooked during preoperative examination and surgical operation as a potential space between the posterior lens capsule and the anterior hyaloid membrane. In our study, we diagnosed a case in which silicone oil accidentally entered BS after vitrectomy and implemented a timely surgical treatment to drain the silicone oil in BS.

## Case presentation

**Fig. 1:**
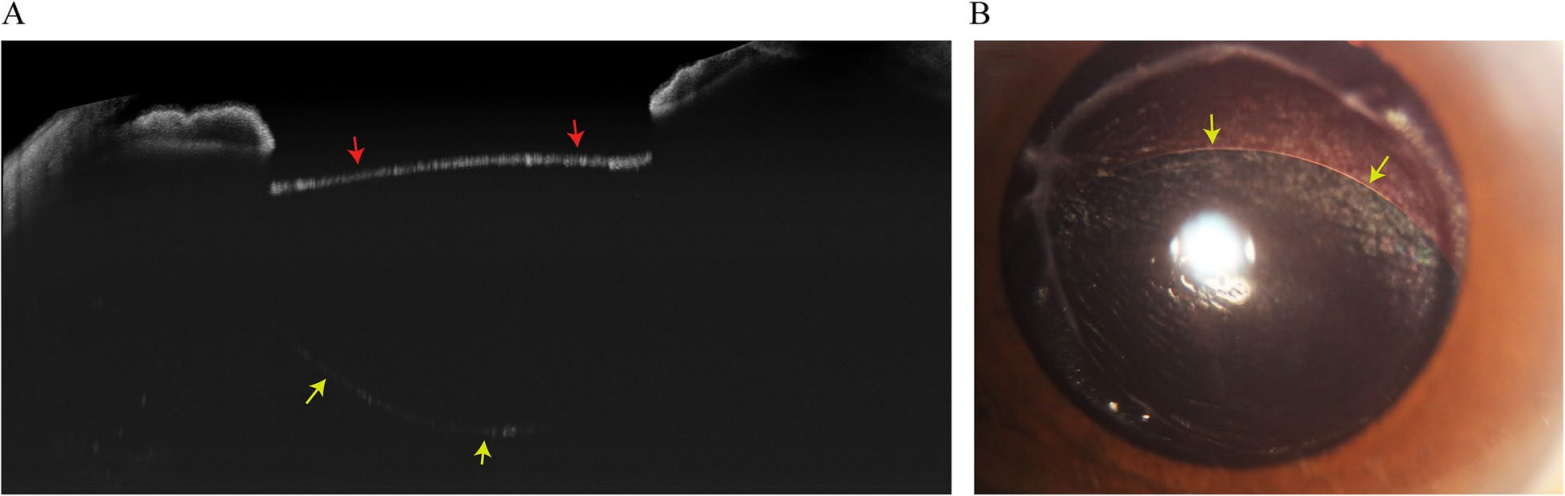
Imaging findings of the patient with a Berger’s space filled by silicone oil. **A** OCT image of the anterior segment shows BS filled by silicon oil. The red arrows indicates the anterior surface of the silicone oil in BS, which is also the posterior lens capsule membrane. The yellow arrows indicates the posterior surface of the silicone oil in BS, which is also the anterior hyaloid membrane. **B** Photographic image of the anterior segment shows BS filled by silicone oil. The yellow arrows indicates the boundary of silicone oil in BS, which is also the boundary of the posterior lens capsule membrane and the anterior hyaloid membrane

A 68-year-old male was diagnosed with right eye retinal detachment (RD) with a history of high myopia, hypertension, and diabetes. During the primary operation, the standard three-port 23-gauge pars plana vitrectomy (PPV) approach and silicone oil injection were implemented after phacoemulsification. Scleral indentation was conducted during PPV to excise the anterior vitreous. At the sixth month follow-up, we noticed an unexpected lens-like round translucent substance located behind the posterior lens capsule of the right eye through the slit lamp examination. The examination of the cornea, aqueous humor, iris, and pupil of the aphakic right eye demonstrated no remarkable signs. The ultrasound and dilated funduscopic examination separately showed the silicone oil-filled vitreous space and flattened retina with high myopia features. Moreover, the anterior optical coherence tomography (OCT) and photographic image of the anterior segment displayed the boundary of silicone oil in Berger’s space, including the posterior lens capsular membrane and the anterior hyaloid membrane, which are shown in Fig. 1. The visual acuity of the patient was HM OD and 20/100 OS. The intraocular pressure was 17 mmHg OD and 17 mmHg OS. The pupillary light responses of both eyes were normal.

In the second surgery, we first conducted the removal of silicone oil in the vitreous space through a vitreous aspiration probe. Afterward, a 23-gauge (G) vitrectomy cutter was used to excise the fibrous wrapping wall of BS, followed by the drainage of silicone oil in BS with the suction of 600 mbar. Finally, the vitrectomy was conducted to completely remove the retained vitreous body. The patient returned 3 months later with the visual acuity of 20/200 OD and 20/63 OS. The intraocular pressure was 15.7 mmHg OD and 12.6 mmHg OS. The lens posterior capsule was clear.

## Discussion and conclusions

The existence of Berger’s space (BS) was discovered as early as the eighteenth century, but few cases of BS have been reported clinically. For instance, Li and colleagues identified BS in a child because of a traumatic hemorrhage accumulated in it [4]. Additionally, Kim et al. reported the dislocation of a posterior chamber phakic intraocular lens in BS in an individual examined post traumatically [5]. Moreover, regarding a patient with diabetic retinopathy (DR), Dubrulle and colleagues reported a stacked dexamethasone implant in BS [6]. In addition to blood and implants, some teams also reported BS with the help of the optical coherence tomography (OCT) technique [1, 7]. In a Belgian study, Tassignon and colleagues offered clear OCT pictures of BS in three patients, among whom one was diagnosed with high myopia [7]. A Spanish team provided OCT (Cirrus Lumera 700 Carl Zeiss Meditec) evidence of BS in three patients. Intriguingly, two of them were also reported to have myopia with -2 diopters and -8 diopters, respectively [1].

To the best of our knowledge, this is the first case reporting BS filled by silicone oil. Due to the difficulties inherent in displaying BS, our report offers a picture of the posterior lens capsule and the anterior hyaloid membrane from a relatively unique perspective. Similar to previous reports, the current patient also had a history of high myopia. We assumed that the pathophysiology of high myopia may contribute to the detachment of the adhesion between the posterior lens capsule and anterior hyaloids, such as the defect on Weiger's ligament and its surrounding tissues. The extension of the axial length may ease the adhesion so that the silicon oil can enter and accumulate in BS. Nonetheless, the existence of BS has been identified in rare cases, whether people with high myopia have a larger BS has not yet been concluded and requires further prospective studies.

There are two conditions under which silicone oil enters BS. First, there should be a complete structure of BS, consisting of an intact posterior lens capsular membrane and an intact anterior hyaloid membrane. Second, there should exist a defect in Weiger's ligament and its surrounding tissues, which enables silicone oil in the vitreous cavity to enter BS. In our case, the patient had a defect in Weiger's ligament and its surrounding tissues, probably caused by high myopia, coupled with our failure to excise the anterior hyaloid membrane completely during the operation, resulting in silicone oil entering BS. Therefore, for patients with high myopia or trauma who have a high risk of a defective Weiger's ligament and its surrounding tissues, clinicians should pay more attention to complete removal of the vitreous anterior cortex and the anterior hyaloid membrane during vitrectomy to avoid such complications.

As an anatomical site linking the anterior and posterior segments of the eye, the knowledge of BS would contribute to our understanding of certain complications that occur in numerous surgeries besides our case. For example, the infusion misdirection syndrome observed in some cataract surgeries may be due to the misdirected flux of aqueous humor into BS [3]. Moreover, the defect on Weiger's ligament caused by blunt trauma provided an avenue by which pigment could reach BS from the posterior chamber, resulting in pigment dispersion syndrome of the central posterior capsule [2]. To date, all the above mentioned reports contain only a small number of cases, and further relevant studies employing larger cohorts are warranted to provide more robust and in-depth findings.

In conclusion, our report is the first case reporting the Berger’s space filled by silicone oil, which is the same as a rare complication of vitrectomy. Furthermore, we explored the possible etiology of silicone oil entering BS, as well as methods for its treatment and prevention. We believe that this case report will provide clinicians with good insights for diagnosis and treatment.

## Supplementary Information

**Additional file 1.** The surgical operation video of the vitrectomy and the silicon oil removal in Berger’s space.

## Data Availability

All data generated or analyzed during this study are included in this published article and its supplementary information files.

## Abbreviations

BS: Berger’s space
RD: Retinal detachment
PPV: Pars plana vitrectomy
OCT: Optical coherence tomography
DR: Diabetic retinopathy

## References

[1] Santos-Bueso E (2019). Espacio de Berger. Arch Soc Esp Oftalmol.

[2] Al-Mezaine HS (2010). Central posterior capsule pigmentation in a patient with pigment dispersion and previous ocular trauma: a case report. Indian J Ophthalmol.

[3] Grzybowski A, Kanclerz P (2018). Acute and chronic fluid misdirection syndrome: pathophysiology and treatment. Graefes Arch Clin Exp Ophthalmol.

[4] Li ST, Yiu EP, Wong AH, Yeung JC, Yu LW (2017). Management of traumatic haemorrhage in the Berger's space of a 4-year-old child. Int Ophthalmol.

[5] Kim JY, Kim KH, Lee JE (2016). Traumatic Dislocation of Posterior Chamber Phakic Intraocular Lens into the Berger's Space. Korean J Ophthalmol.

[6] Dubrulle P, Fajnkuchen F, Qu L, Giocanti-Aurégan A (2016). Dexamethasone implant confined in Berger's space. Springerplus.

[7] Tassignon MJ, S ND. Real-Time Intraoperative Optical Coherence Tomography Imaging Confirms Older Concepts About the Berger Space. Ophthalmic Res. 2016;56(4):222–6. 10.1159/000446242 27352381

